# Building a search tool for compositely annotated entities using Transformer-based approach: Case study in Biosimulation Model Search Engine (BMSE)

**DOI:** 10.12688/f1000research.128982.1

**Published:** 2023-02-10

**Authors:** Yuda Munarko, Anand Rampadarath, David Nickerson

**Affiliations:** 1 Auckland Bioengineering Institute, University of Auckland, Auckland, 1010, New Zealand; 2 The New Zealand Institute for Plant and Food Research Limited, Auckland, New Zealand

**Keywords:** Transformer, BERT, biosimulation model search engine, semantic annotation, CASBERT, Physiome Model Repository, composite annotation, information retrieval

## Abstract

The Transformer-based approaches to solving natural language processing (NLP) tasks such as BERT and GPT are gaining popularity due to their ability to achieve high performance. These approaches benefit from using enormous data sizes to create pre-trained models and the ability to understand the context of words in a sentence. Their use in the information retrieval domain is thought to increase effectiveness and efficiency. This paper demonstrates a BERT-based method (CASBERT) implementation to build a search tool over data annotated compositely using ontologies. The data was a collection of biosimulation models written using the CellML standard in the Physiome Model Repository (PMR). A biosimulation model structurally consists of basic entities of constants and variables that construct higher-level entities such as components, reactions, and the model. Finding these entities specific to their level is beneficial for various purposes regarding variable reuse, experiment setup, and model audit. Initially, we created embeddings representing compositely-annotated entities for constant and variable search (lowest level entity). Then, these low-level entity embeddings were vertically and efficiently combined to create higher-level entity embeddings to search components, models, images, and simulation setups. Our approach was general, so it can be used to create search tools with other data semantically annotated with ontologies - biosimulation models encoded in the SBML format, for example. Our tool is named Biosimulation Model Search Engine (BMSE).

## Introduction

Many natural language processing (NLP) tasks have seen significant performance improvements since the introduction of the Transformer ^1^ and the derived technology such as BERT ^2^ and GPT. ^3^^,^^4^ The Transformer is an encoder-decoder structure and was originally designed for natural multi-language translation. Its advantages lie in the attention mechanism and position coding to memorise long source sentences and carry out training efficiently in parallel. Then, GPT explores the decoder section by creating pre-training models from extensive data using unsupervised learning to further fine-tune the models for specific NLP tasks using supervised learning. BERT works on a similar fine-tune concept but specifically uses the encoder section to train in parallel and recognise the context of words bidirectionally. These positive attributes draw our attention to implement the Transformer, specifically BERT, in developing a search tool for data compositely annotated using ontologies.

Biosimulation models are examples of data that are largely annotated using ontologies, *e.g.* those written using the CellML ^5^^,^^6^ and Systems Biology Markup Language (SBML) ^7^ standards. Structurally, the biosimulation model is composed of constant or variable that form more complex entities such as mathematical equations, components or reactions and in overall represent the model. To give expressive and complete descriptions, it is recommended to provide semantic annotations even compositely with multiple ontology instances. ^8^ Now, entities can be rediscovered for various purposes, such as new model composition, initial variable search, and model verification. Furthermore, this way of annotation is encouraged by the Computational Modeling in BIology NETwork (COMBINE) community as a standard to ensure interoperability and sharing between different platforms. ^9^^,^^10^

SPARQL helps to find entities and has been recommended as a standard by W3 Community. However, creating SPARQL is difficult as it requires good knowledge of annotation structure, syntax, and ontology terms. Tools to help generating SPARQL from natural languages, such as NLIMED, ^11^ are helpful. Still, it is more suitable for experts who already know the target entity, not for those doing exploratory searches like using commercial search engines. Furthermore, annotations are mainly for the lowest and highest entities; as in biosimulation models, there are rarely component and image annotations. Consequently, finding these unannotated entities using SPARQL is impossible.

BERT is a cross-encoder that takes a pair of sentences as input and generates a classification embedding and a set of token embeddings. The classification embedding is used to calculate the probability that the last sentence is a continuation of the first sentence. Although the implementation for information retrieval has high accuracy, its efficiency is low because it has to generate embeddings of pairs of a query to all sentences. Reducing the number of embedding creations, several studies have used BERT as a re-ranker by initially retrieving sentences using traditional methods such as bag of word; the top n results are considered as relevant. ^12^^–^^14^ However, the formation of one embedding that represents each sentence will be more convenient, resulting in more efficient computation and simpler data management. BERT can receive a sentence and then output token embeddings. The average of token embedding is considered as a sentence embedding. Still, the results are not satisfactory, because the BERT paradigm is a cross-encoder for the next sentence prediction task. Therefore, Sentence-BERT implements a siamese architecture to train language models using BERT so it can generate sentence embedding. ^15^ Sentence-BERT is a bi-encoder that does not preserve the query context in the target sentence. It is much more efficient, although its effectiveness may be lower than other approaches implementing a cross-encoder. The poly-encoder modify BERT to maximise performance by accommodating both encoders and tweaking the cross-encoder. ^16^ Although it is more efficient than original BERT, it is still not comparable to Sentence-BERT. Then, ColBERT uses a polling approach by calculating the similarity of each query token embedding with each sentence token embedding. ^17^ The maximum scores are taken and then summed, and the sentences with the higher total score are given a higher rank. Due to the simplicity of Sentence-BERT, Composite Annotation Search Using BERT (CASBERT) uses it to represent ontology classes and predicates in composite annotations. ^18^ The composite annotations are converted into embeddings representing entities in biosimulation models.

In this paper, we demonstrate the practical use of a Transformer-based approach to build a search tool on a biosimulation model stored in the Physiome Model Repository (PMR) ^19^ using CASBERT. There are five entity levels in ascending order: variables/constants, components, models (CellML), images, and simulation setups (SED-ML). Initially, we created embeddings for variables/constants where most of the composite annotations are available. Variables/constants are the fundamental entities that make up the components and further form the model, while at the same time, images and simulation setups can be equivalent to models. Therefore, combining embeddings from the lowest to the highest level will form embeddings at each entity level. The availability of text-based annotations that are unique to an entity, such as the model authors and filename, are also useful to make the embedding more distinctive. Further, we also used these text-based annotations to create high-level entity embedding whose constituent entities are not annotated. Now the entity search simply converts the query to an embedding using CASBERT and then calculates its similarity against embeddings organised by level and displays the results in order. The search tool we created is named Biosimulation Model Search Engine (BMSE).

Accessing SBML models from the BioModels database ^20^ to BMSE is possible due to the similar annotation procedures and data formats, and this will be our future work. Other domains with semantic annotations such as gene experiments in the ArrayExpress ^21^ and food microbiology models in ComBase ^22^ could similarly implement our approach in their searching tools.

Our implementation, dataset, indexes, and source code are publicly available [ 1].

## Methods

We developed a search tool to find entities in the PMR ^19^ organised by entity levels. The PMR primarily consists of biosimulation models encoded in the CellML format. ^5^^,^^6^ Composite annotations explaining entities in the CellML were extracted and converted to entity embeddings using CASBERT. ^18^ Then, entity embeddings were managed by their levels in a list-like structure to simplify the creation, replacement, update, and deletion (CRUD) processes. Our tools are built in a modular manner to improve reusability and to help aid sustainability by making maintenance easier.

### CellML

CellML is a standard format for encoding mathematical models related to biological systems. ^5^ Structurally, a model is composed of components which are the smallest units that can be reused to form other models. ^23^ The component contains one or more mathematical equations composed of variables or constants. Then, all these entities together with supplemental or derived entities, *e.g.* schematic diagrams or simulation plots, are needed for various purposes such as checking initial values of variables, reuse of constants, model composition, and reproduction of experiments. Here, we used entities consisting of 4652 variables/constants, 1987 components, 980 models, 295 simulation setups and 980 images. Our approach allows for inexpensive addition and modification of entities and will be described in the next subsection.

**Figure 1.**
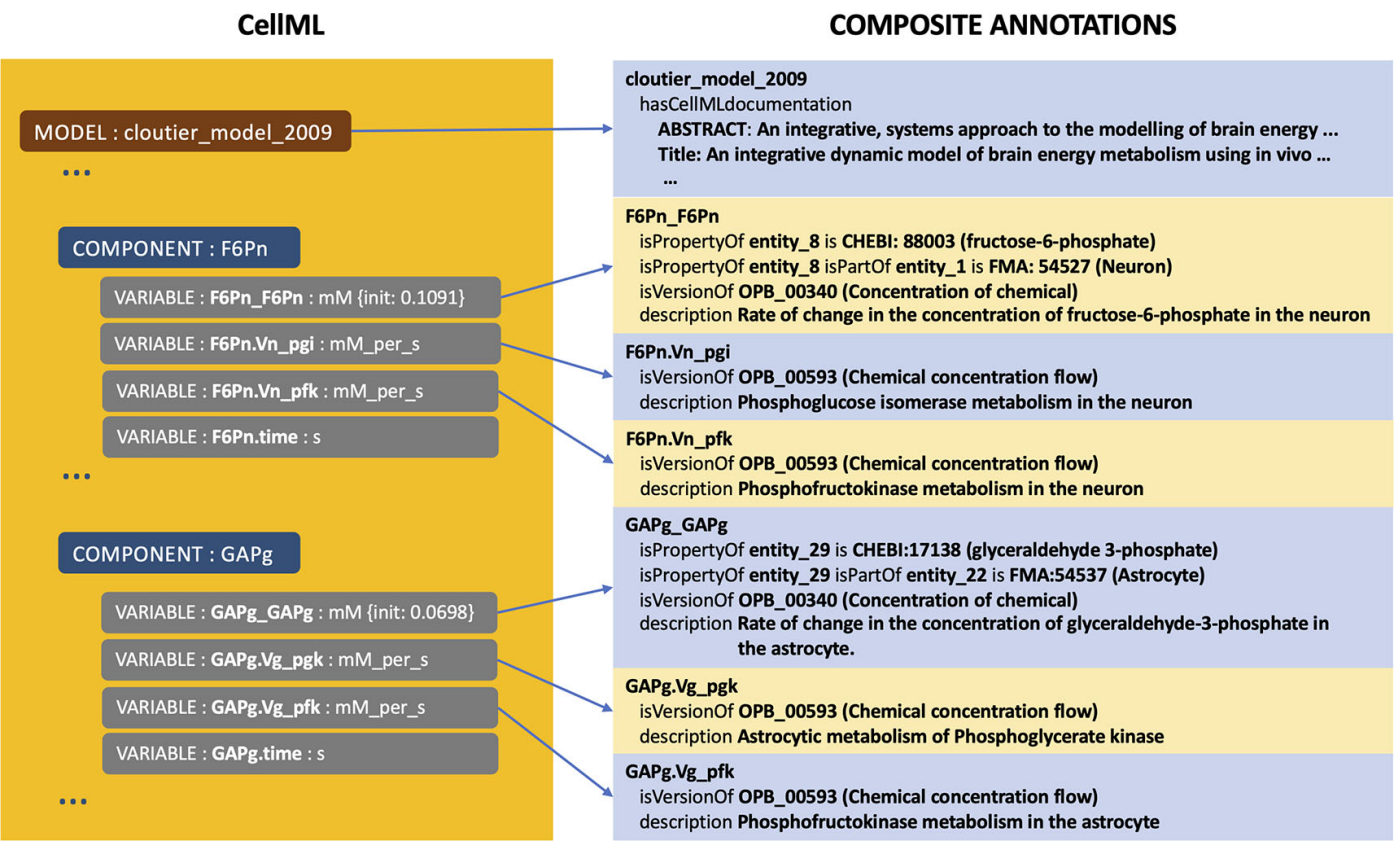
The example of an CellML file with its entities, including models, components, and variables, along with composite annotations describing the models and variables. The model is about brain energy metabolism; the two components, GAPg and F6Pn, are related to glyceraldehyde-3-phosphate and fructose-6-phosphate, consecutively. ^24^

Figure 1 shows the example of composite annotations in the model of brain energy metabolism ^24^ encoded using CellML model available in the PMR [ 2]. There are two components, GAPg and F6Pn, where each component having four variables, and structurally is similar. Concerning GAPg, three variables, *i.e.* GAPg_GAPg, GAPg.Vg_Pgk, and GAPg.Vg_Pfk, are annotated to Ontology of Physics for Biology (OPB), ^25^ Foundational Model of Anatomy (FMA), ^26^ and Chemical Entities of Biological Interest (ChEBI). ^27^ GAPg_GAPg is the main variable and is the output of the component, providing information about the rate of glyceryldehyd-3-phosphate concentration change in an astrocyte. To give a complete and expressive description, this variable is compositely annotated with OPB_00340:Concentration of chemical, FMA:54537:Astrocytes, and CHEBI:17138:glyceryldehyd-3-phosphate complete with relationship predicates such as isVersionOf, isProperyOf, and isPartOf. Other variables are the input arguments obtained from the other components and annotated with OPB_00593: Chemical concentration flow. A similar annotation pattern is performed for most other CellML files where primarily at the variable/constant entities. At the same time, other annotations are mainly for model entities as descriptive metadata such as title, abstract, and author (see Figure 1).

### CASBERT

We used CASBERT to create variable/constant embeding. CASBERT ^18^ is a tool for converting the composite annotation of an entity into an embedding by applying Sentence-BERT. ^15^ Sentence-BERT is used to convert textual properties related to ontology classes ( *e.g.* CHEBI:17138, OPB_00340) and predicates ( *e.g.* isPropertyOf, isVersionOf) to embeddings. Then, the embeddings is merged to create an embedding representing variable/constant.

Technically CASBERT can implement other approaches such as ColBERT ^17^ and poly-encoder ^16^ with the increase in computational and indexing complexity; the use of Sentence-BERT is preferred because of its practicality while still providing good performance. Other alternatives are Onto2Vec ^28^ and OPA2Vec ^29^ which use Word2Vec ^30^ to translate the words in the ontology class to embeddings, and then merge them as an ontology embedding. However, a word embedding approach only considers word co-occurrence in the training data, so the embedding of a particular word will be the same for all sentences. Moreover, it does not consider the context of the sentence.

### Hierarchical search for biosimulation models

Here we describe our approach to build a search tool for biosimulation models encoded using CellML in the PMR. The tools consists of hierarchical entity embedding collection where the entity embeddings are organised based on their level. Query evaluation was carried out to determine the ranking of entities according to level based on their similarity to the query.

**Hierarchical entity embedding collection**

**Figure 2.**
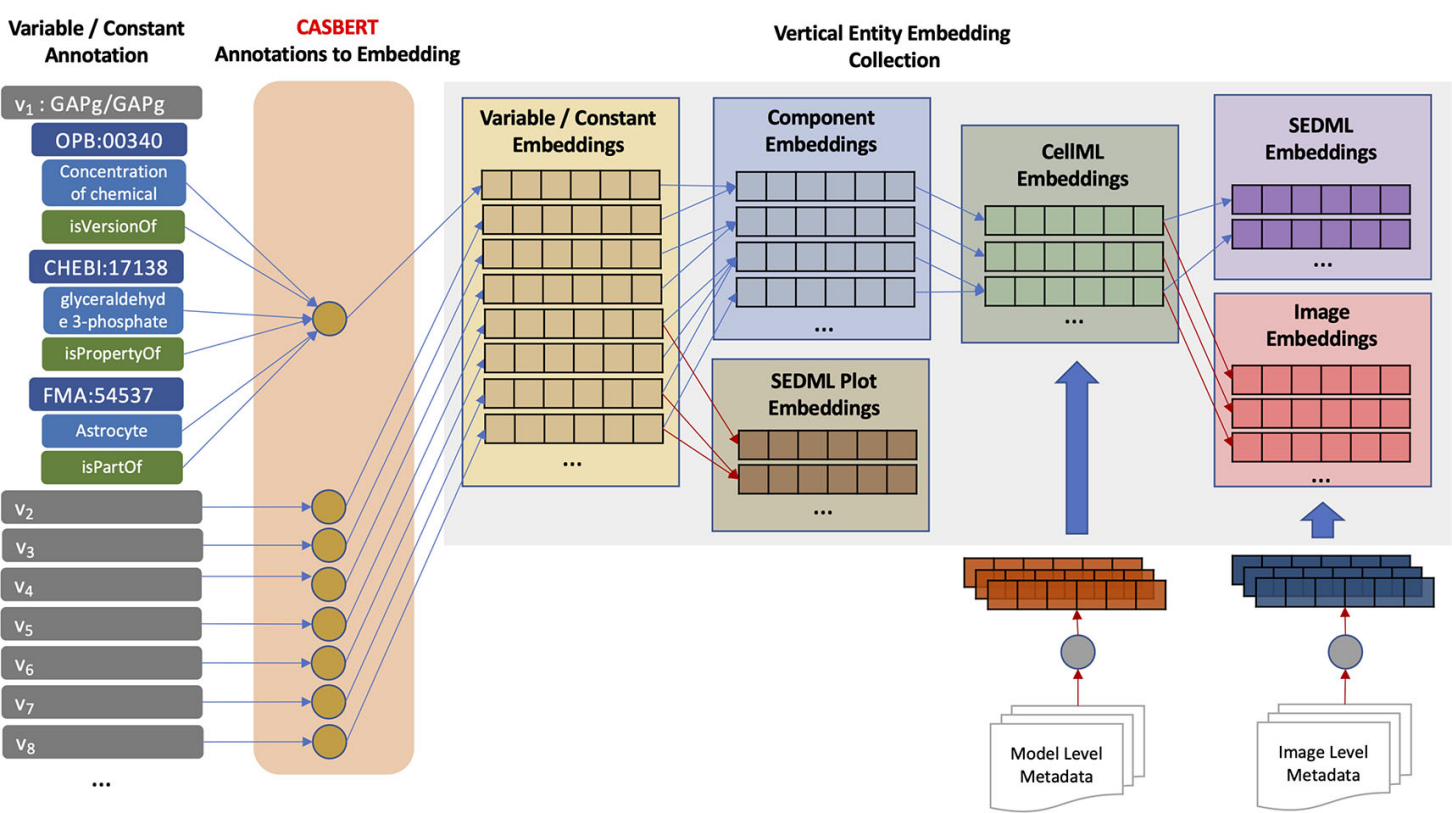
The process of creating a hierarchical entity embedding collection starts by converting the composite annotations of variables/constants, continues with components, then SED-ML plots and models (CellML), and finally, simulation setups (SED-ML) and images.

Entity embeddings are grouped vertically, and collectively called a hierarchical entity embedding collection. With this grouping, users can search for entities specific to their level and possibly get a better searching experience. Figure 2 shows the process of creating the collection, starting from the lowest-level entity, variable/constant, and then combined to gradually create higher-level entity embeddings, *i.e.* constant, model (CellML), simulation setup (SED-ML), and image. Initially, CASBERT converts the composite annotations of the variables/components, resulting in the variable/constant embeddings. Then, one or more variable/constant embeddings are combined to form component embedding. Combining embeddings can be done by concatenation or merging. Concatenation can represent the combined embedding well, while merging may reduce information. However, merging is advantageous for the uniform embedding size, making it easier to measure similarity. Moreover, Coates and Bollegala ^31^ demonstrated that merging by averaging could retain most information because high-dimensional embeddings are considered nearly orthogonal; therefore, we used this approach. We implemented Equation 1 for averaging by removing duplication and dividing the sum of embeddings *E* by the number of embeddings | *E*|. Using this averaging, SED-ML plot embeddings were also created; nevertheless, the generated entities are few and therefore, BMSE did not include them.

$$h=\frac{\sum E}{\left|E\right|},\text{where}\;E=\left\{e_{1},\ldots ,e_{n}\right\}$$ (1)

**Figure 3.**
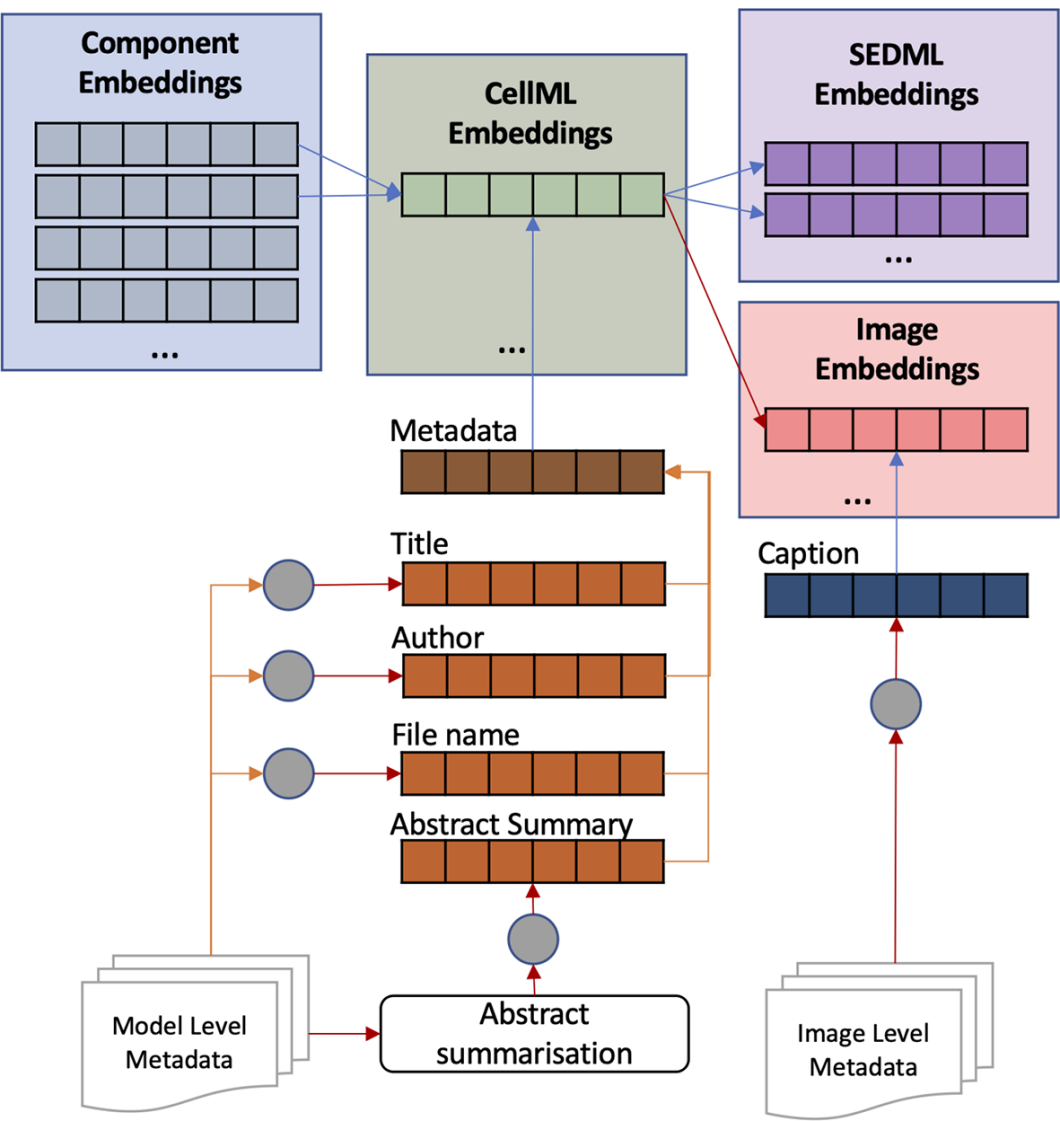
The process of combining embeddings to create model (CellML), simulation setup (SED-ML), and image embeddings. Model and image embeddings are enriched with metadata embeddings. The metadata includes title, author, filename, abstract summary, and image caption.

Model (CellML) embeddings are created based on the component embeddings and additional metadata describing the model. The metadata mostly contains the reference article information such as filename, title, authors, and abstract. The short metadata, *i.e.* filename, title, and authors, are converted directly to embeddings; in contrast, the long metadata, *i.e.* abstract, is summarised first by taking the important phrases using SciSpacy. ^32^ These phrases are put together as one text and then converted into embedding. The summarisation is to overcome the BERT input limit of 512 tokens. Tokens are generated using WordPiece ^33^ so abstracts with a maximum of 250 words will likely have more than 512 tokens. Then, all metadata embeddings are merged, and the result is combined with the merged component embeddings, all using Equation 1 (see Figure 3). Image and simulation setup (SED-ML) embeddings are the same as model embeddings; however, an image usually has a caption. Therefore, the image embedding is a combination of caption embedding and model embedding. More than half of CellML files in the PMR are not annotated, leading to incomplete results when searching on model semantics alone. With metadata embeddings, most unannotated models can be represented.

**Query evaluation**

The query used to search for entities is converted to embedding using CASBERT. The process in CASBERT involves both macro embedding and micro embedding. Macro embedding is the entire query text converted to embedding, whereas micro embedding is a combination of phrase embeddings. Then the addition of macro and micro embeddings forms a query embedding.

**Figure 4.**
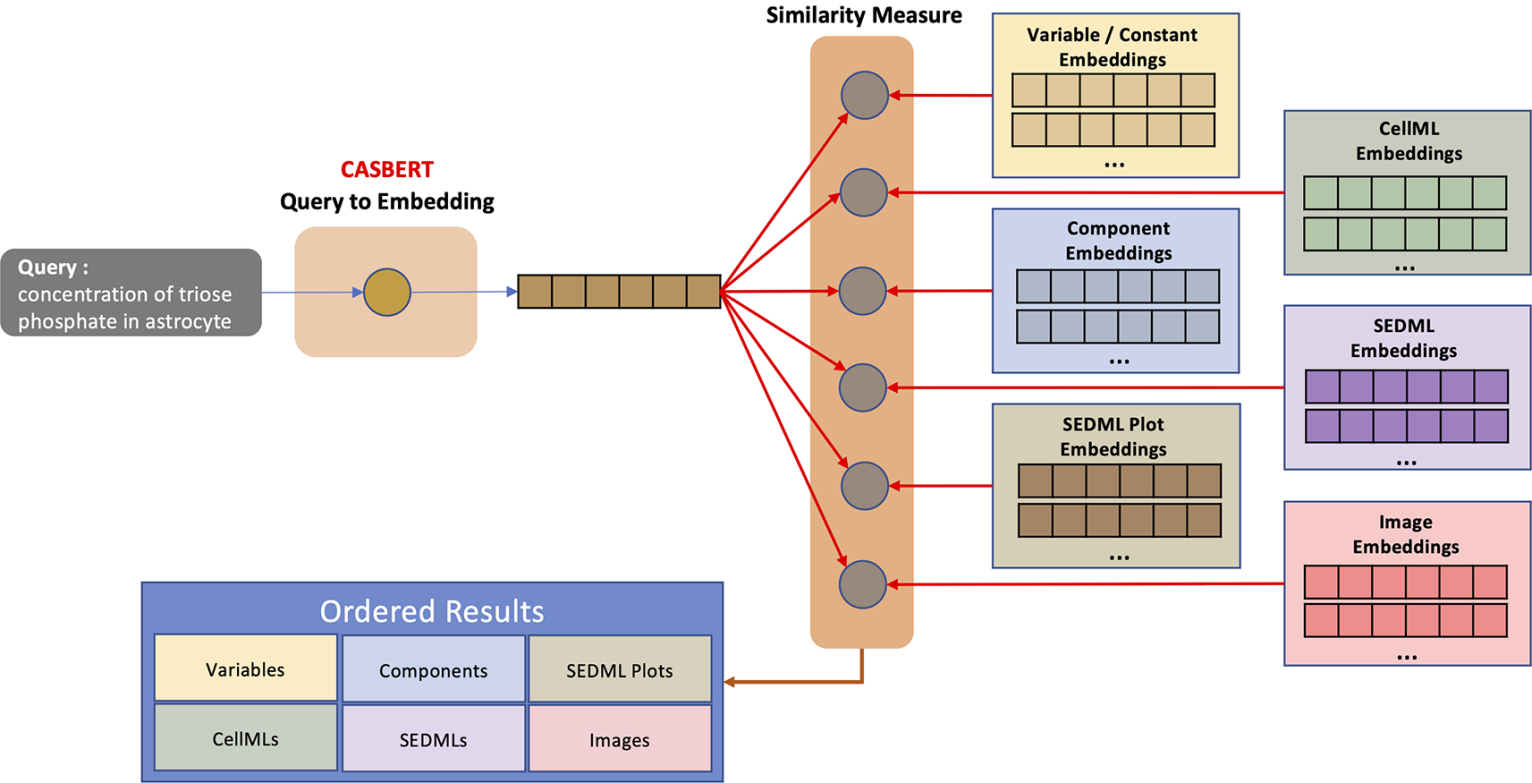
Query evaluation to compare a query to the hierarchical entity embedding collection. The query is converted to embedding using CASBERT; then, the similarity is measured against the embedded entities at each level. The results are presented in order and displayed by level.

Figure 4 shows the hierarchical entity search process, where a query embedding is measured against each entity embedding at each hierarchical level. The measurement uses cosine similarity, ^34^ which is suitable for non-normalised Sentence-BERT-based embedding. The results are displayed based on the hierarchical levels, so it helps users to find entities by types, such as initial values for variables, reaction formulas, and model images.

### BMSE implementation

Hierarchical entity embedding collection enables modular data management and we further developed the search tool in a modular fashion. This modularity speeds up the development, deployment, and maintenance processes where the modules created have a consistent interface, so algorithm and data changes do not affect other modules.

**Data organisation**

We defined the embeddings and other related data for each entity level in EntityEmbeddings and EntityData objects, respectively. The EntityEmbeddings is a two-dimensional tensor created using PyTorch ^35^ where each element is an embedding representing an entity. This tensor, along with PyTorch, is efficient in calculating and sorting the similarities of query embedding to each entity embedding. Data organisation, *i.e.* CRUD, are quite fast because the tensor data format is simple and resembles a list. CRUD operation in the high entity level should not affect the lower level but not the other way around. To facilitate entities propagation when there is a CRUD operation in lower level entity, pointers to the higher level entities are encoded in EntityData. In addition to pointers, EntityData stores other information according to its level, for example at the variable/constant level there are initial values and equations, while at the model level there are workspace links and other related models. Furthermore, the EntityEmbeddings and EntityData of each entity level are stored in the form of files so that they are easy to implement and deploy on different platforms.

**Modular development**

BMSE modularity is generally divided into frontend and backend. The frontend implements a JavaScript Framework providing an interface for interactive use of BMSE in the web browser. Users can search for entities by keywords; then, with the search results, they can perform advanced activities such as comparing entities, searching for images and dependent equations, and copying LaTeX code for equations. Results presented by the frontend are provided by the backend performing tasks such as query processing, similarity measurement, entity retrieval, and data formatting. Accessing the backend is standardised, as are the return formats, so there is a guarantee that changes to the backend do not interfere with the frontend. Regarding the CRUD processes of EntityEmbeddings and EntityData, the backend will not be affected as the processes can be done without shutting down the service.

**Model cluster**

We found a large number of structurally similar models in the PMR due to model composition and modification. They differ in completeness of information, *e.g.* images, titles, annotations, and some low-level entity details. Sharing information in such a model can provide a more comprehensive description. To do this, we extracted three types of structures from each CellML file, namely the XML document as a whole (all-structures), the entity as a whole (deep-structure), and the path from the entity root to the leaf (wide-structure). Then, these structures are converted into a term frequency (TF) and Inverse Document Frequency (IDF) matrix and finally clustered using hdbscan. ^36^

### Operation

BMSE requires Docker 20.10.x for deployment on a local machine or cloud instance. The local machine does not need specific specifications; however, the cloud instance should be Linux based, where we use Ubuntu, with 4 VCPUs and 8GB RAM. We provide the BMSE pipeline in the GitHub repository [ 3] and the archive in Zenodo [ 4].

## Experiments and results

In this section, we present Biosimulation Model Search Engine (BMSE), a web-based search tool, to find model entities (variables/constants, components, models, simulation setups, and images) in the Physiome Model Repository (PMR). Queries in BMSE are keywords consisting of phrases, abbreviations, and formulas, so it provides flexibility of search expression and effortless refinement.

### Entity discovery

Users search for entities by submitting queries as keywords, and then BMSE presents results organised by level. Using keywords allows users to access information just like in a commercial search engine. Keywords are structured intuitively and can be modified by generalisation, specification or reformulation. Later, users can customise the results, such as displaying at a certain level only, sorting by attributes, and filtering based on ontology classes. The customised results then are ready for knowledge extraction, *e.g.*, the variable initial values, simulation plots, entity comparison, and model authors. Hence, different information needs may lead to different workflows which users freely describe.

**Figure 5.**
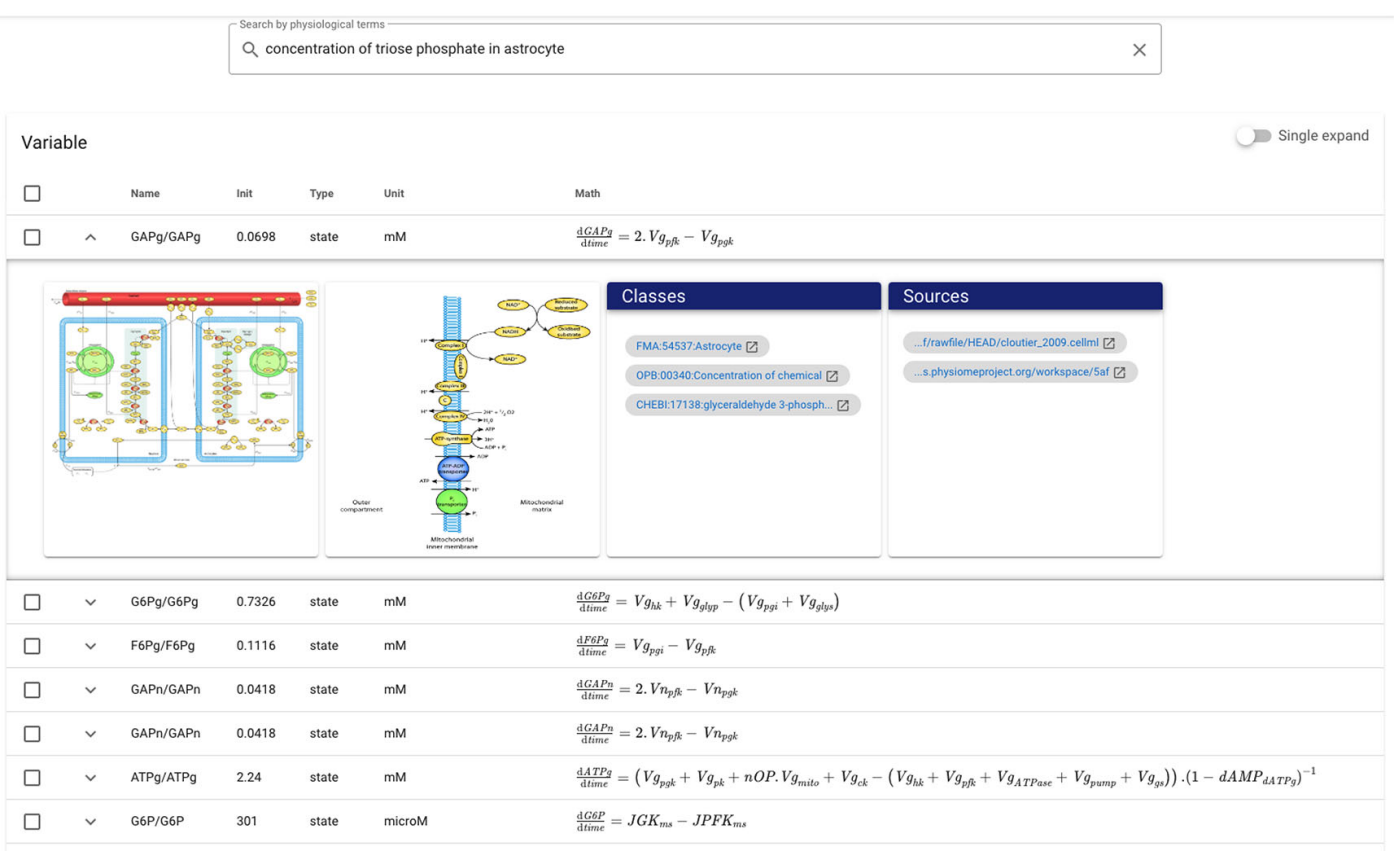
The example of a search for variable/constant entities with the keywords ‘concentration of triose phosphate in astrocytes’. The results show entities with variable initial values, types, units, and mathematical equations. Each entity is expandable to get more detailed information such as related images, models, and ontology classes.

Figure 5 is an example of the results at the variable/component level for the query ‘concentration of triose phosphate in astrocytes’ where triose phosphate is the synonym of glyceryldehyd-3-phosphate. Variable/constant entities are arranged based on similarity values and are described with the name, initial value, type, unit, and mathematical equation attributes. For the query example, GAPg/GAPg with glyceryldehyd-3-phosphate is correctly presented in the top position, followed by G6Pg/G6Pg with Robison ester, both of which are about the chemical concentration in astrocytes. Expanding entities can access detailed information about these chemical compounds, images, and models. At the component level, the top-ranking entities are usually those containing the top variables at the variable/constant level. Most components present mathematical equations representing the variables that carry out the process or reaction. The results at the model level are quite different, where models with higher ranking variables or components can be in a lower order. However, this makes sense because the sample query is suitable for low-level entity search. Models can contain many variables and components with various annotations; thus, queries require more general keywords such as article authors and title. Simulation setup and image levels follow the model level. The simulation setup shows the resulting plot with the initial values of the associated variables. Due to caption embedding, image level results may differ slightly from model level.

### Entity comparison

Biosimulation models in the PMR are mainly based on published articles. These articles may discuss the same object with different assumptions and approaches and present new biosimulation models combining the available models. Therefore, there are many similar entities at all levels; they differ in the content of particular attributes such as initial variable values, formulas, authors, and approaches used. BMSE provides an interface to compare entities and spot differences so that users can select the suitable entities.

**Figure 6.**
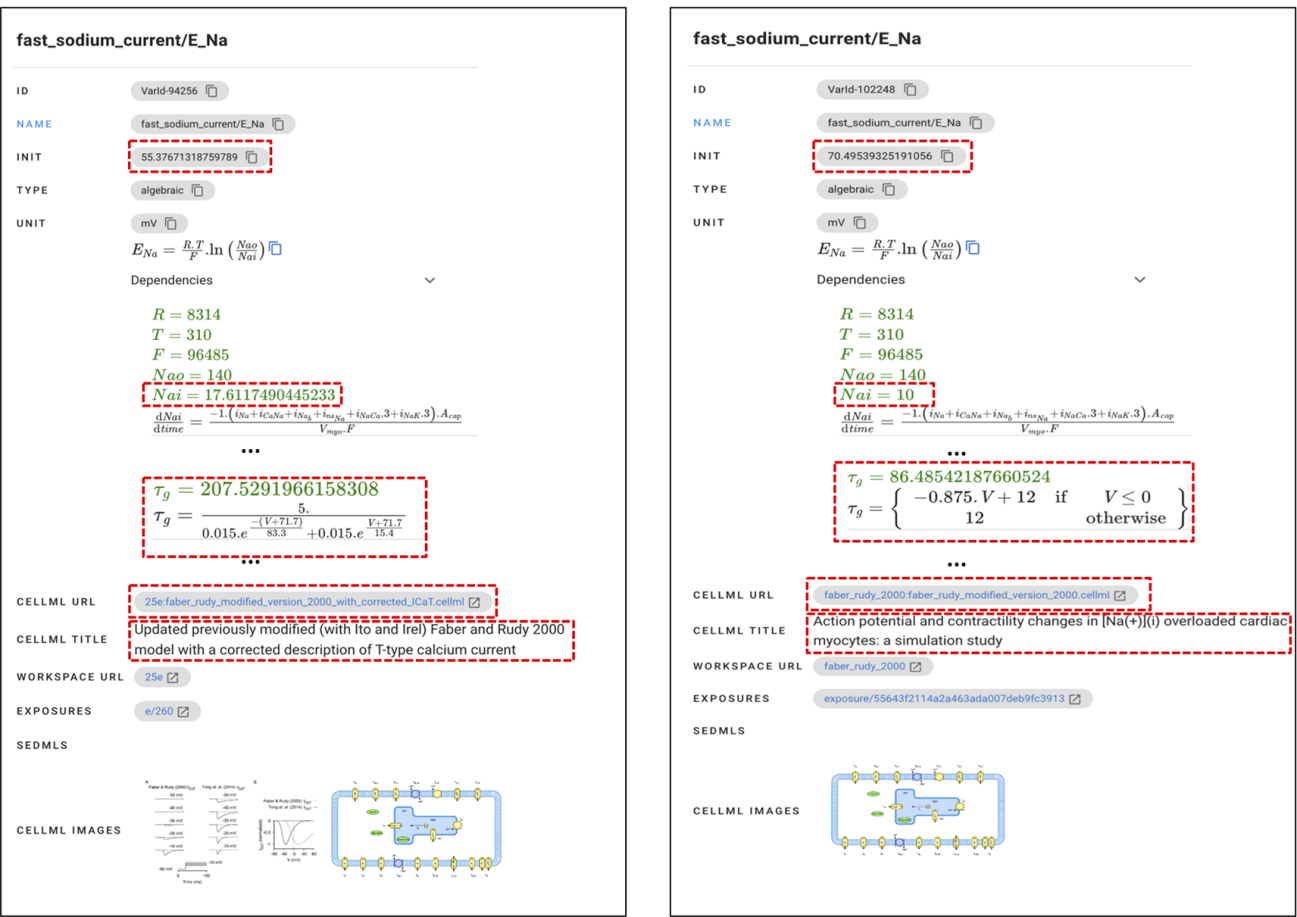
The interface in BMSE showing the comparison of variable *E _Na_* from Ref. 41 but with a different initial values. Further differences are analysed to select the one suitable for user needs.

Suppose a user is looking for the initial value of voltage-gated sodium channels and then makes a query ‘sodium channel voltage’. Of the entities shown, the variable *E _Na_* in the *fast_sodium_current* component is the suitable one and is described with the voltage-gated sodium channel activity (GO:0005248) and complex (GO:0001518). However, there are nine variations of *E _Na_* initial value, all of them with the same mathematical formula, type, and units, found in models from five articles. ^37^^–^^41^ Even the same variable from the same article may have a different initial value such as 62.748 mV, 62.904 mV, 70.495 mV, and 55.377 mV in Ref. 41. The user can further analyse these variations by comparing the model, image, and mathematical equation attributes (see Figure 6) and then selecting one of the initial values.

### Entity reuse

Now with BMSE, it is possible to reuse entities over a broader range of physiology. Entities in different models can be found and presented together with the relevant information. Tools such as the Epithelial Modeling Platform (EMP) ^42^^,^^43^ can use the BMSE web service to explore candidate entities and ensure their compatibility quickly and accurately. The EMP is used to assemble a new model using public entities, and compatibility checks are essential in guaranteeing a plausible new model. For the *E _Na_* variable in the previous subsection, for example, if the assembled model is mammalian with sodium overload-induced, the variable in Ref. 41 might be the most appropriate.

## Discussion

We have demonstrated a practical approach to building a search tool using a Transformer-based approach for compositely annotation entities in models in the PMR. Here we discuss the advantages of using Transformer and the potential use of the developed search tool (BMSE). The last subsection examines limitations and future works for BMSE.

### The advantage of using transformer

Our approach to using CASBERT offers the organisation of entity embeddings vertically and modularly. Creating embeddings is incremental from the lowest to the highest level, so it is simple and fast. Then, the embeddings are organised in a simple data structure, assuring efficient management without compromising system performance when performing CRUD operations. Each embedding at different levels is arranged in a modular manner, allowing for the application of modular designs when developing the tool. While not mandatory, separating data, models, and views is suitable for future continuous tool development.

### The use of BMSE

In the biosimulation model community, there are standards and tools to describe models and entities using composite annotations, *e.g.*, SemGen. ^44^ The tools make the annotation consistent, and consequently the number of the annotated entities are increased. The consistent annotation was demonstrated to be valuable for new model compositions such as in the Epithelial Modelling Platform (EMP) ^43^ and bond-graph based hierarchical composition. ^45^ Here, BMSE can be an intermediary tool serving entities for further reuse, and facilitate the FAIR data principles in the biosimulation model domain.

### Implementation for other domains

We see the potential use of our approach in other domains by applying semantic annotations such as the experimental gene datasets in ArrayExpress ^21^ and predictive models of food microbiology in the ComBase ^22^ repositories. Our approach is designed to accommodate composite annotations and hierarchical entities but is not mandatory. In the absence of hierarchical entities, entities can also be grouped according to specific criteria independently as needed. ArrayExpress is now equipped with Expression Atlas, ^46^ an interface for exploring experimental results, which displays information organised by organism, anatomy, and gene expression. The query is assisted with the autocomplete and suggestion features. While the results are sophisticated, we thought our approach would help enable more expressive queries.

We use CASBERT, which is Transformer-based as well as embedding-based. As an alternative, our approach allows for adapting other embedding systems such as word-based, ^30^^,^^47^^,^^48^ GPT-based, ^3^^,^^4^ and other BERT-based. ^15^^–^^17^ In the future, we could rapidly implement a new embedding-based system with better performance; this is an essential side of our practice.

### Limitations and future works

We identify limitations in our work that, when done, may improve performance and user experience; this will be our future work. The Sentence-BERT model for converting text to embedding has not been fine-tuned for models in the PMR. Fine-tuning could use ontology classes involved in the composite annotation or the entire ontologies. We expect that the fine-tuned model will allow queries using more general terms not used for existing annotations in the PMR, such as ‘macroglial’ to find entities with either of the more specific terms ‘astrocytes’ or ‘oligodendrocytes’.

We suggest that lower-level entity embedding should also store information about higher-level entity embedding, just as variable/constant embedding should contain information about its model. This hold will allow, for example, to search for variable/constant entities by the article author. Combining higher-level entity embedding with lower-level embedding can use a weighted average. However, this is not covered here as we focus on the process of developing hierarchical entity embeddings.

Currently, BMSE does not consider user intent and only treats queries equally against all entity levels. By identifying the user intent, we could apply entity-level selection to the query (vertical selection) and customise the results by aggregating information from multiple entities (aggregate view). To achieve this, however, data about user behaviour in searches on BMSE is required, which is currently unavailable. In future, the data could be collected as user query logs and analysed to find correlations between queries and information needs. Finally, the search is expected to accommodate natural language in the form of questions and answers.

Extending BMSE to integrate with model repositories including content similar to the PMR ( *e.g.*, the BioModels database) is possible due to the harmonised manner for annotating models compositely. The combination of these repositories provides the most extensive biosimulation model collection supporting a more comprehensive range of entity reuse and robust model verification. By integrating suitable translation tools, such as Tellurium ^49^ for CellML to SBML and SBML to CellML translation, it is possible to envision extending BMSE to guide the reuse of models independent of the source model repository.

## Conclusions

We have presented the use of a Transformer-based approach in building a retrieval tool for data annotated compositely. In this case, we used biosimulation models encoded with CellML standard deposited in the Physiome Model Repository (PMR). Each model is arranged hierarchically from the variable/constant entities to the model entity. Our efficient approach can construct embeddings representing these entities and organise them in a simple data structure. Therefore, this makes it possible to implement the same strategy in other domains. Our searching tool is named Biosimulation Model Search Engine (BMSE). People in the biosimulation model community can explore entities specific to their level, *i.e.* variable/constant, component, model, simulation setup, and image. Finding these entities is crucial for the following activities: model composition, verification, and reproduction, therefore, supporting the FAIR data principles. In this paper, we presented the necessary steps to develop the tool and have identified some opportunities for future developments.

## Data Availability

• The PMR models indexed by BMSE: https://models.physiomeproject.org/
License: Attribution 3.0 Unported (CC BY 3.0) • The PMR model used as an example in this manuscript: https://models.physiomeproject.org/workspace/5af/
License: Attribution 3.0 Unported (CC BY 3.0) • The method used, CASBERT, to convert queries and entities to embeddings and search entities: https://github.com/napakalas/casbert/ ( https://doi.org/10.5281/zenodo.7549557). ^50^
• BMSE documentation and tutorials to implement and reproduce query examples: Figshare: BMSE Documentation and Tutorials, https://doi.org/10.17608/k6.auckland.21679394.v1

## Footnotes

^1^

https://github.com/napakalas/bmse/

^2^

https://models.physiomeproject.org/workspace/5af/

^3^

https://github.com/napakalas/bmse/

^4^

https://doi.org/10.5281/zenodo.7549557

## Software availability

Source code available from: https://github.com/napakalas/bmse/tree/v.1.0.0

Archived source code at time of publication: https://doi.org/10.5281/zenodo.7549557. ^50^

License: GNU GPL-3.0
