# Supplementary material for: Building a search tool for compositely annotated entities using Transformer-based approach: Case study in Biosimulation Model Search Engine (BMSE)
Source: F1000Res. 2023 Feb 10;12:162. [Version 1] doi: 10.12688/f1000research.128982.1 (PMC10570691; doi:10.12688/f1000research.128982.1)
Supplement: Peer review history

# Reviewer report: Reviewer response for version 1

**DOI:** 10.5256/f1000research.141628.r205266

**Published:** 2023-10-12

**Authors:** Garima Malik

**Affiliations:** 1 Toronto Metropolitan University, Toronto, Ontario, Canada

***Summary of research:***

 The authors developed a search tool called CASBERT using BERT technology. This tool helps search through data annotated using ontologies. They used biosimulation models encoded with CellML standard from the Physiome Model Repository (PMR) as their dataset. The tool can highlight various entities such as variables/constants, components, models, simulation setups, and images. It also creates embeddings for these entities and organizes them in a simple data structure.This article provides detailed technical information on building the tool, including the GitHub code. To enhance the article, my minor suggestions for improvement are outlined below.

***Suggestions:***

- The article could provide more detailed insights into the applications of the word embeddings generated by the tool.
- The introduction lacks a smooth flow in language.
- Additionally, it's important to explicitly state the contributions of the research at the end of the introduction section.
- The results section lacks discussion on metrics, which are crucial to validate the effectiveness of the proposed tool.

Are the conclusions about the tool and its performance adequately supported by the findings presented in the article?

Partly

Is the rationale for developing the new software tool clearly explained?

Yes

Is the description of the software tool technically sound?

Yes

Are sufficient details of the code, methods and analysis (if applicable) provided to allow replication of the software development and its use by others?

Yes

Is sufficient information provided to allow interpretation of the expected output datasets and any results generated using the tool?

Yes

Reviewer Expertise:

Natural Language Processing (NLP), Machine Learning and Large Language Models (LLMs), Weak Supervision, Data Programming.

I confirm that I have read this submission and believe that I have an appropriate level of expertise to confirm that it is of an acceptable scientific standard.

# Reviewer report: Reviewer response for version 1

**DOI:** 10.5256/f1000research.141628.r163793

**Published:** 2023-03-10

**Authors:** Socrates Dokos

**Affiliations:** 1 Graduate School of Biomedical Engineering, University of New South Wales, Sydney, NSW, Australia

This article describes a search tool for biological computational models in the Physiome Model Repository based on a Composite Annotation Search Using BERT (CASBERT) Transformer approach. The main advantage of the approach is that composite annotations can be efficiently searched for in entities ranging from author/title, variables, model components, images to simulation setups. The article is well-written: I have only minor typographical corrections to suggest, as per below.

 Minor Corrections

- Page 3, paragraph 2: replace “composed of constant or variable” with “composed of constants or variables”.
- Page 3, paragraph 3: replace “by W3 Community” with “by the W3 Community”.
- Page 3, paragraph 4: replace “The poly-encoder modify BERT” with “The poly-encoder modifiesBERT”.
- Page 4, paragraph 5: delete “where” from “A similar annotation pattern is performed for most other CellML files were primarily at the variable/constant entities”.
- Page 4, paragraph 6: replace “Then, the embeddings is merged to create an embedding representing variable/constant” with “Then, the embeddings are merged to create an embedding representing avariable/constant”.
- Page 4, paragraph 8: replace “The tools consists of hierarchical entity embedding collection” with “The tools consists of a hierarchical entity embedding collection”.
- Page 5, figure 1 caption: replace “an CellML file” with “a CellML file” and “consecutively” with “respectively”.
- Page 9, paragraph 3: replace “for compositely annotation entities in models in the PMR” with “for compositely-annotated entities in models in the PMR”.
- Page 10, figure 6 caption: replace “but with a different initial values” with “but with different initial values”. Also, replace “user needs” with “user need”.
- Page 10, paragraph 3: replace “and other BERT-based” with “and other BERT-based approaches”.
- Page 10, paragraph 4: replace “but with a different initial values” with “but with different initial values”. Also, replace “user needs” with “user need”.
- Page 10, paragraph 3: replace “We identify limitations in our work that, when done” with “We identify limitations in our work that, when addressed”.

Are the conclusions about the tool and its performance adequately supported by the findings presented in the article?

Yes

Is the rationale for developing the new software tool clearly explained?

Yes

Is the description of the software tool technically sound?

Yes

Are sufficient details of the code, methods and analysis (if applicable) provided to allow replication of the software development and its use by others?

Yes

Is sufficient information provided to allow interpretation of the expected output datasets and any results generated using the tool?

Yes

Reviewer Expertise:

Computational modelling of biological systems, neuroscience, electrophysiology, biomedical engineering

I confirm that I have read this submission and believe that I have an appropriate level of expertise to confirm that it is of an acceptable scientific standard.
